# A prospective, randomized, triple-blind comparison of articaine and bupivacaine for maxillary infiltrations

**DOI:** 10.4317/medoral.17476

**Published:** 2011-12-06

**Authors:** Miguel A. Vílchez-Pérez, Manuel Sancho-Puchades, Eduard Valmaseda-Castellón, Jordi Paredes-García, Leonardo Berini-Aytés, Cosme Gay-Escoda

**Affiliations:** 1 DDS. Fellow of Oral Surgery and Implantology, School of Dentistry, University of Barcelona (Spain); 2 DDS. Fellow of Oral Surgery and Implantology, School of Dentistry, University of Barcelona (Spain); 3 PhD. Professor of Oral and Maxillofacial Surgery, Master’s Degree Program in Oral Surgery and Implantology, School of Dentistry, University of Barcelona, Barcelona, Spain. Researcher of the IDIBELL Institute; 4 DDS, MD. Professor of the Master’s Degree Program in Oral Surgery and Implantology, School of Dentistry, University of Barcelona, Barcelona, Spain. Researcher of the IDIBELL Institute; 5 DDS, MD, PhD. Dean, Professor of Oral and Maxillofacial Surgery, Master’s Degree Program in Oral Surgery and Implantology, School of Dentistry, University of Barcelona, Barcelona, Spain. Researcher of the IDIBELL Institute; 6 DDS, MD, PhD. Chairman and Professor of Oral and Maxillofacial Surgery. Director of the Master of Oral Surgery and Implantology. School of Dentistry of the University of Barcelona. Coordinator/Researcher of the IDIBELL Institute. Head of the Oral and Maxillofacial Surgery Department of the Teknon Medical Center, Barcelona (Spain)

## Abstract

Objectives: To compare the clinical anesthetic efficacy of 0.5% bupivacaine and 4% articaine (both with 1:200.000 adrenaline) for anterior maxillary infiltration in healthy volunteers. Material and methods: A triple-blind split-mouth randomized clinical trial was carried out in 20 volunteers. A supraperiosteal buccal injection of 0.9 ml of either solution at the apex of the lateral incisor was done in 2 appointments separated 2 weeks apart. The following outcome variables were measured: latency time, anesthetic efficacy (dental pulp, keratinized gingiva, alveolar mucosa and upper lip mucosa and tissue) and the duration of anesthetic effect. Hemodynamic parameters were monitored during the procedure. Results: Latency time recorded was similar for both anesthetic solutions (p>0.05). No statistically significant differences were found in terms of anesthetic efficacy for dental pulp, keratinized gingiva or alveolar mucosa. Articaine had a significant higher proportion of successful anesthesia at 10 minutes after infiltration in lip mucosa and lip skin (p=0.039). The duration of anesthesia was 336 minutes for bupivacaine and 167 minutes for articaine. (p<0.001). No significant hemodynamic alterations were noted during the procedure. Conclusions: Articaine and bupivacaine exhibited similar anesthetic efficacy for maxillary infiltrations. The duration of anesthesia was longer with the bupivacaine solution, but lip anesthesia was better with articaine.

** Key words:**Articaine, bupivacaine, maxillary, infiltrative anesthesia, long-acting anesthetics.

## Introduction

Bupivacaine is a long-acting local anesthetic that has been marketed for dental use since 1971. Various studies have been published evaluating the clinical efficacy of bupivacaine, and although it does not differ much chemically from mepivacaine, another anesthetic from the same group, bupivacaine has clearly distinct clinical properties. Compared with other local anesthetics, the main advantages of bupivacaine are longer anesthetic effect and prolonged residual analgesia. The main concern is its potential cardiovascular toxicity (1).

Articaine was synthesized in 1969, but it was not until 1976 that it would be introduced for clinical dentistry. It was first marketed in Germany and subsequently throughout Europe and Canada, and in 2000, in the United States. It has some unique chemical characteristics: it is the only amide local anesthetic that contains a thiophene ring and no aromatic ring. Besides, it is the only widely used amide local anesthetic that contains an additional ester ring within its chemical structure (1-3).

Several reports (4-8) have evaluated and compared bupivacaine versus other local anesthetics, especially for lower third molar removal. However, few studies comparing bupivacaine and articaine have been published, the present study being the first clinical trial comparing these two anesthetics for maxillary infiltrations.

The aim of this work was to compare the clinical anesthetic efficacy of 0.5% bupivacaine and 4% articaine, both with 1:200.000 adrenaline for maxillary infiltrations. A secondary objective was to detect any hemodynamic alterations after the anesthetic injection.

## Material and Methods

A triple-blind randomized controlled clinical trial (RCT) was conducted in healthy student volunteers at the Dental School of the University of Barcelona (Spain) from July 2009 to January 2010. Participants were recruited using advertisements describing the objective of the study, the inclusion criteria and payment for participation. The research protocol was according the Helsinki’s declaration and was approved by the institutional review board (Ethics Committee for Clinical Investigation of the Dental School of the University of Barcelona). The clinical trial was conducted following the guidelines of the CONSORT statement (9). The inclusion criteria were healthy volunteers (ASA I) between 18 to 30 years old; with absence systemic disease, no background of medication hypersensitivity or pregnancy; no toxic habits (including alcohol abuse, smoking or regular cannabis smoking or other drug use); absence of routine medication use; absence of adverse reaction to local anesthetics; absence of dental diseases (tooth decay or other abnormalities), tooth restorations, traumatic lesions, dental hypersensitivity or periodontal disease for all teeth under study; positive pulp vitality tests in all teeth under study; absence of acute or chronic infections in the oral and maxillofacial area.

The exclusion criteria were use of any medication for 15 days prior to study; use of local anesthetics in the oral and maxillofacial area for 15 days prior to study; heart rate lower than 50 or higher than 90 beats/min; latency time longer than 3 minutes during infiltration. In this case, infiltration is repeated in another session to rule out the possibility of errors in anesthesia administration; if a volunteer drops out.

The RCT had a split-mouth, crossover design. Randomization was based on a sequence generated by Laboratorios Inibsa, Barcelona, Spain. Both solutions were encoded so that the surgeon performing the anesthesia infiltration, the monitor recording the variables and the volunteer could not identify the anesthetic solution used.

At the first appointment, we completed a thorough anamnesis and clinical examination and checked for fulfilment of inclusion criteria. We provided an explanation of details of the study and patients signed an informed consent form.

During the second appointment, infiltrative anesthesia was performed on an upper lateral incisor with the contralateral canine as the control. At the third appointment sides were changed: anesthesia of the upper lateral incisor not selected during the second appointment and contralateral canine as the control. The same surgeon (MAVP) performed all anesthetic infiltrations and the outcome variables and hemodynamic values were registered by the same examiner (MSP). Both appointments were spaced at least 2 weeks apart.

Anesthetic solutions under study were bupivacaine 0.5% and articaine 4%, both with 1:200.000 adrenaline, and were specifically prepared by Laboratorios Inibsa, Barcelona, Spain. For anesthesia infiltration we used a Uniject® syringe (Laboratorios Normon, Madrid, Spain) with a short 27G Monoprotect XL® needle of 25 mm length (Laboratorios Inibsa, Barcelona, Spain). Volunteers were placed in the supine position with chest at an angle of 30 degrees. After negative aspiration was performed, 0.9 mL of the anesthetic solution was deposited over a 30-seconds time period.

-Outcome variables

Latency time: it was subjectively assessed by the first reported sensation of upper lip numbness. We classified the time interval between needle removal and numbness onset in 3 categories: immediate after needle removal, after less than 30 seconds or after 30 seconds or more.

Soft tissue anesthesia: we determined anesthetic efficacy on the attached gingiva, alveolar mucosa upper lip mucosa and lip skin with a sharp 4/6 Maillefer exploratory probe (Dentsply Maillefer, Tulsa, USA). We considered anesthesia effective when patient did not notice any pain when the exploratory probe was pressed with a light force 3 consecutive times. In case of pain or doubtful answer, anesthetic efficacy was judged to be a failure. The values were recorded at 5, 10, 15, 20, 25, 30, 35, 40, 50 and 60 minutes after anesthesia infiltration.

Pulpal anesthesia: We determined the pulp vitality using the Elements Diagnostic Unit and Apex Locator (SybronEndo Corporation, Orange, USA). During the first appointment, baseline information was obtained for both lateral incisors and upper canines. On the 2 subsequent visits, pulpal anesthesia of the anesthetized lateral incisors was assessed together using the contralateral canines as a negative control. We considered pulpal anesthesia successful if electric pulp tester readings were higher than 60 without eliciting pain. Values were recorded at 5, 10, 15, 20, 25, 30, 35, 40, 50 and 60 minutes after anesthesia infiltration.

Total duration of anesthesia (soft tissues): time of infiltration injection was recorded for each procedure. The duration of lip numbness was assessed by training the subjects to report when soft tissue anesthesia wore off and register the time. We registered the time interval between anesthetic infiltration and cessation of subjective sensation of lip numbness.

The patient was assessed during 60 minutes. After, patients were discharged and were instructed to report the time the anesthesia had worn off.

-Hemodynamic parameters

Baseline hemodynamic parameters (heart rate, systolic blood pressure, diastolic blood pressure and oxygen saturation) were recorded during the first appointment using a Guardian BPM-730 M monitor (Megos-Sonmédica, Barcelona, Spain). On the 2 subsequent visits, they were assessed 1 minute before and 1 minute after anesthesia infiltration, and then at 5, 15, 30 and 60 minutes after infiltration.

-Statistical analysis

A descriptive, bivariate statistical analysis was performed using the SPSS version 14.0 statistical package (SPSS; SPSS Inc; Chicago, USA). Intrasubject variability was compared by Student’s t-test, McNemar’s test and Analysis of Variance (ANOVA). Intersubject variability was assessed by Student’s t-test and Spearman’s test. Statistical significance value was set as p<0.05.

## Results

**Figure 1:**
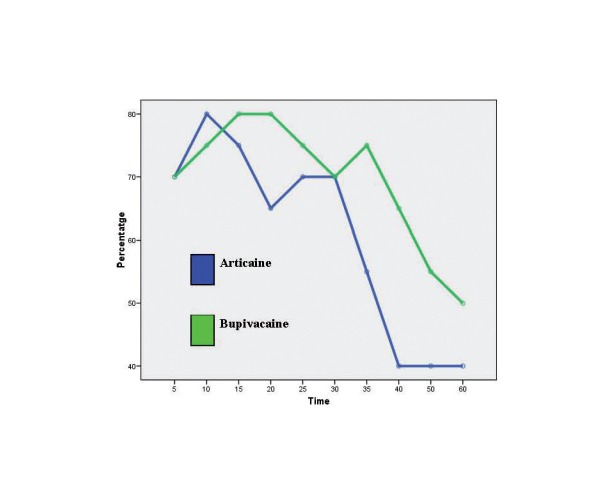
Onset of pulpa anesthesia.

A total of 33 volunteers agreed to participate in the study. Seven were excluded as they were allergic to some medication, 2 because of negative responses obtained for pulp vitality test for some teeth under study and other 4 for incompatibility with work hours (Fig. 1). The total sample was 20 volunteers (15 women and 5 men) with a mean age of 22.75 year (SD=2.15). Hemodynamic parameters recorded during the whole procedure were within the standard values (Table  Table 1). No statistically significant differences were found in terms of systolic blood pressure, diastolic blood pressure, heart rate and oxygen saturation for both anesthetic solutions during the intervals under study (ANOVA test p>0.05). No complications connected with the anesthesia administration were reported.

**Table 1:**
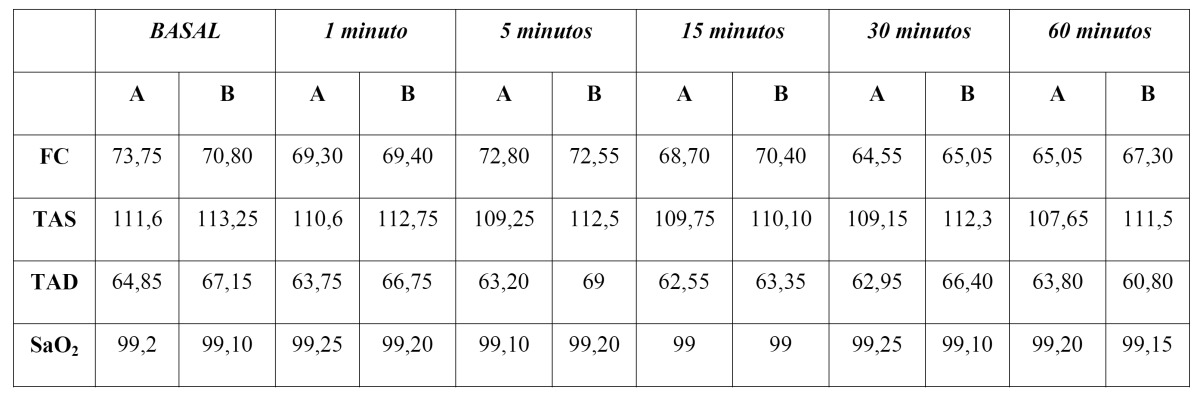
Hemodynamic parameters recorded at each time of examination. Column A is the articaine solution and column B to the solution with Bupivacaine. HR = heart rate (beats / min), SBP = systolic blood pressure (mmHg), DBP = diastolic blood pressure (mmHg), SaO2 = oxygen saturation (%).

Latency time for soft tissue anesthesia in both anesthetic solutions did not show statistically significant differences. In the bupivacaine group, 80% volunteers reported that anesthesia was felt before withdrawing the needle 10% a period shorter than 30 seconds and the remaining 10% longer than 30 seconds. Articaine showed similar results as 85% felt numbness before withdrawal of the needle, 10% described a period shorter than 30 seconds and 5% longer than 30 seconds.

Both solutions had a similar efficacy for pulpal anesthesia. In the two groups, 100% success rate of anesthetic efficacy was not obtained during the procedure. Bupivacaine seemed to be effective in more cases from minute 15 until minute 60, but these differences were not significant (Fig. 1).

**Figure 2:**
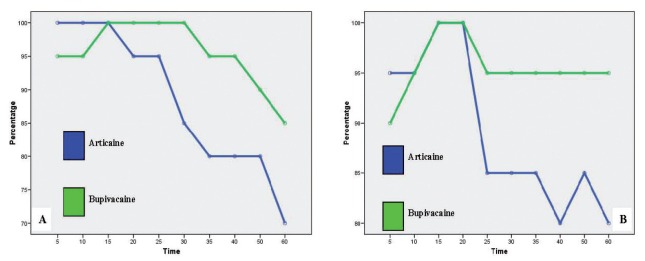
A) Anesthesia efficacy on keratinized gingiva. B) Anesthesia efficacy on alveolar mucosa.

The anesthesia administration on keratinized gingiva and alveolar mucosa did not report statistically significant differences for both solutions. As well as in pulpal anesthesia, bupivacaine showed a greater anesthetic efficacy compared with articaine, from minute 15 (keratinized gingiva) and minute 20 (alveolar mucosa) until the end of procedure (Fig. 2).

**Figure 3:**
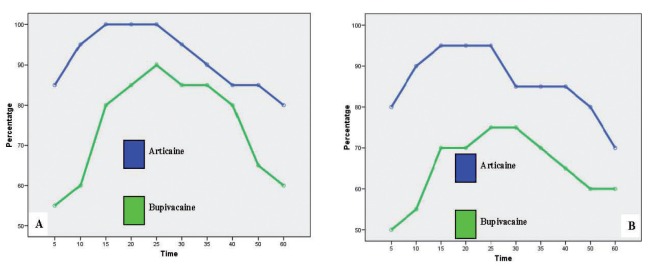
A) Anesthesia efficacy on lip mucosa. B) Anesthesia efficacy on upper akin.

In contrast, lip mucosa did report statistically significant differences at 10 minutes after infiltration (Fig. 3). At that moment, anesthetic efficacy of articaine was 95% and bupivacaine only 60% for the volunteers reporting effective anesthesia (McNemar’s test p=0.039). Similar differences were also found for lip skin. Articaine proved greater anesthetic efficacy (90%) at 10 minutes compared with bupivacaine (55%) (McNemar’s test p=0.039). At 15 and 20 minutes after anesthesia infiltration, differences were obtained for both anesthetic solutions, although they were no statistically significant (p=0.063). For the lip mucosa and lip skin, articaine exhibited greater efficacy during the whole procedure (Fig. 3).

Total duration of anesthesia (soft tissues) reported by participants was 336.00 minutes (SD=166.46) for bupivacaine, whereas articaine obtained lower results, with a mean duration of 163.45 minutes (SD=57.48). Differences found were statistically significant (t-Student p=0.000).

## Discussion

Latency time, anesthetic efficacy and duration of anesthesia are the main parameters studied in other reports that use a similar study design. However, it is difficult to compare results as dosages, anesthetics, use of vasoconstrictors and methods differ (10).

Latency time was calculated as the interval between injection and the time when anesthesia is achieved. It represents a key factor when choosing the anesthetic solution. It can be determined using different calculations and will vary according to the areas under study or treatment. In the present study, the latency time was subjectively evaluated by participants who reported upper lip numbness. Our results are similar to those obtained by Kennedy et al. (11) who reported a latency time of 0.86 minutes for 0.5% ropivacaine used for lip anesthesia (assessed as well according to the onset of lip numbness). Gross et al. (12) did also evaluate the anesthetic efficacy of 0.5% bupivacaine with 1:200.000 epinephrine in maxillary lateral incisors. The result, though, was 3.19 minutes (SD=3.72) as results were obtained for pulpal anesthesia. According to Evans et al. (13), the onset of pulpal anesthesia was 2.5 minutes (SD=1.22) in maxillary lateral incisors and 3 minutes (SD=1) for Vähätalo et al. (14), although adrenaline concentrations were different:1:100.000 and 1:200.000 respectively.

The main difference between our study and earlier works (11-14) is that we determined the efficacy of soft tissue anesthesia as well as pulpal anesthesia. Thus, we were able to find differences in both anesthetic solutions that could go undetected otherwise. Furthermore, the use of a triple-blind, split-mouth, RCT design optimized sample size. In addition, the administration of just 0.9 mL of anesthetic solution helped to reduce the duration of anesthesia.

With regards the evaluation of pulpal anesthesia, Dreven et al. (15), and Certosimo and Archer (16) concluded that pulpal anesthesia was successful when readings of 80 were obtained from the electric pulp tester because that lower readings were associated with pain during restoration. This criterion was not been included in our study for ethical reasons, as all participants were healthy volunteers, anesthesic dosage was low (0.9 mL) and the main objective was to compare the efficacy of both solutions with no need for further treatment. For this reason, a 60 reading obtained by the pulp tester was considered enough.

No statistically significant differences were found in the present study for both solutions used for anesthesia of dental pulp, keratinized gingiva and alveolar mucosa. Besides, no statistical differences were found compared with other authors in terms of anesthetic efficacy achieved at 5 minutes after infiltration. In our study, bupivacaine showed 70% of pulpal anesthesia at 5 minutes, whereas Gross et al. (12) the success of the infiltration of the bupivacaine solution was 78% and according to Kennedy et al. (11), 80%. A lower result may be associated with the use of a lower dose of anesthetic solution (0.9 mL). Articaine showed same result at 5 minutes, 70%. Evans et al. (13) reported a result of 88%. It is important to underline that such percentages may vary depending on the methodology used for analysis and the use of different anesthetic solutions. For example, Gross et al. (12) reported that the proportion of successful anesthesia in lateral incisors after maxillary infiltration with lidocaine solution was 97%, while Evans et al. (13), using a similar methodology reported only a 62%.

When comparing the success rate for lip mucosa and lip skin, articaine reported higher results during the whole procedure. However, bupivacaine did not show similar or greater results. Anesthetic technique involved periapical supraperiostic infiltration of upper lateral incisor, thus anesthetic effect on lip mucosa and lip skin should be regarded as the consequence of anesthetic solution diffusion to the areas far away from infiltration area. The fact the bupivacaine did not offer similar success rates to those obtained by articaine and the statistical differences found at minute 10 may be connected with the results reported by some authors (6,17-19), as bupivacaine has a low diffusion rate into soft tissues. Because bupivacaine has high protein binding, the above results may be justified and latency time may be prolonged as the diffusion into the infiltrated area may be delayed.

Furthermore, the high protein binding of bupivacaine may be responsible for a prolonged anesthesia on soft tissues that varies from 6-12 hours. In our study, bupivacaine reported a duration of anesthesia time of 336 minutes (SD=166.46) whereas articaine showed lower results, with a mean time of 163.45 (SD=57.48). Kennedy et al. (11) reported a duration of lip anesthesia of 512.25 minutes (SD=179.76) while Gross et al. (12) obtained results similar to ours, with a duration of 383 minutes (SD=169).

The use of bupivacaine as well as the rest of local anesthetics is not free of risks. In the present study, no significant between both solutions have been found in terms of hemodynamic parameters. Besides, no adverse reactions associated with the use of bupivacaine have been reported. However, the amount of anesthetic solution was 0.9 mL for young, healthy individuals and therefore with low risk of cardiovascular complications. Although several deaths have been associated with the use of bupivacaine, most of such cases referred were not dental treatments and the dosages used were higher than the ones widely used in Dentistry.

Bacsik et al. (4) published a comprehensive review of the literature in order to determine the toxicity from anesthetics such as bupivacaine. They reported that the total dosage that can be administered is lower compared to the rest of local anesthetics belonging to the amide group. However, the low dosage will not substantially change the final number of cartridges that can be used, as concentration for the rest of amide local anesthetics is higher (articaine 4%, lidocaine 2%, mepivacaine 3%) compared with bupivacaine 0.5%.

In conclusion, the anesthetic efficacy obtained for both solutions in maxillary infiltration in terms of pulpal anesthesia, keratinized gingiva and alveolar mucosa is similar. However, lip mucosa was better anesthetized with articaine, suggesting that difussion of bupivacaine could be worse. The concentration and dose used for both solutions did not produce any noticeable hemodynamic alterations.
